# Role of Position 627 of PB2 and the Multibasic Cleavage Site of the Hemagglutinin in the Virulence of H5N1 Avian Influenza Virus in Chickens and Ducks

**DOI:** 10.1371/journal.pone.0030960

**Published:** 2012-02-21

**Authors:** Karel A. Schat, John Bingham, Jeff M. Butler, Li-Mei Chen, Sue Lowther, Tamsyn M. Crowley, Robert J. Moore, Ruben O. Donis, John W. Lowenthal

**Affiliations:** 1 Department of Microbiology and Immunology, College of Veterinary Medicine, Cornell University, Ithaca, New York, United States of America; 2 Commonwealth Scientific and Industrial Research Organisation, Livestock Industries, Australian Animal Health Laboratory, Geelong, Victoria, Australia; 3 Influenza Division, Centers for Disease Control and Prevention, Atlanta, Georgia, United States of America; 4 Centre for Biotechnology, Chemistry and Systems Biology, Deakin University, Geelong, Victoria, Australia; The University of Hong Kong, China

## Abstract

Highly pathogenic H5N1 avian influenza viruses have caused major disease outbreaks in domestic and free-living birds with transmission to humans resulting in 59% mortality amongst 564 cases. The mutation of the amino acid at position 627 of the viral polymerase basic-2 protein (PB2) from glutamic acid (E) in avian isolates to lysine (K) in human isolates is frequently found, but it is not known if this change affects the fitness and pathogenicity of the virus in birds. We show here that horizontal transmission of A/Vietnam/1203/2004 H5N1 (VN/1203) virus in chickens and ducks was not affected by the change of K to E at PB2-627. All chickens died between 21 to 48 hours post infection (pi), while 70% of the ducks survived infection. Virus replication was detected in chickens within 12 hours pi and reached peak titers in spleen, lung and brain between 18 to 24 hours for both viruses. Viral antigen in chickens was predominantly in the endothelium, while in ducks it was present in multiple cell types, including neurons, myocardium, skeletal muscle and connective tissues. Virus replicated to a high titer in chicken thrombocytes and caused upregulation of TLR3 and several cell adhesion molecules, which may explain the rapid virus dissemination and location of viral antigen in endothelium. Virus replication in ducks reached peak values between 2 and 4 days pi in spleen, lung and brain tissues and in contrast to infection in chickens, thrombocytes were not involved. In addition, infection of chickens with low pathogenic VN/1203 caused neuropathology, with E at position PB2-627 causing significantly higher infection rates than K, indicating that it enhances virulence in chickens.

## Introduction

Since 2003, highly pathogenic (HP) H5N1 avian influenza viruses (AIV) of the Asian lineage have caused major outbreaks in ducks, swans and geese and other free-living birds as well as in backyard flocks of chickens and ducks and commercial poultry [1]. By early-2012, H5N1 viruses have infected around 578 humans almost entirely by direct contact with infected birds resulting in a case fatality rate of approximately 59% [2].

Several virus-specific factors seem important in successful interspecies transmission of H5N1 from birds to humans [3]. In particular, functional adaptation of the viral ribonucleoprotein complex appears to be significant since sequence analysis of H5N1 isolates from humans and other mammals indicate that it is under considerable positive selection [4]–[7]. One of the changes in the polymerase basic-2 protein (PB2) protein commonly associated with adaptation of a bird-derived HP H5N1 virus to mammalian species is the change of glutamic acid (E) to lysine (K) at position 627 [8]–[10]. Estimates of the importance of the E to K change depend on the experimental model used. In the mouse model, PB2-627K is significantly more pathogenic than PB2-627E whereas in ferrets there was little difference. Not all human H5N1 isolates from lethal cases have the E to K substitution. Although in the absence of PB2-627K, the change in PB2-701 from D to N change is frequently present [11], and has also been associated with transmission from avian to mammalian hosts [12]. The importance and function of the substitution of E to K for the *in vivo* pathogenicity has not been fully elucidated. Hatta *et al.* [8] examined the pathogenicity of human isolates with PB2-627K obtained from the 1997 Hong Kong outbreak and found that the change of K to E attenuated the infection in mice. The PB2-627K virus spread faster than PB2-627E in infected mice, perhaps overwhelming the host defense [13]. Furthermore, selection for PB2-627K and PB2-701N occurs in humans infected with avian-origin viruses [14]. Similarly, *in vitro* studies showed that the influenza polymerase complex requires K, rather than E, for efficient activity in primate cells [15], [16].

The threat to public health posed by H5N1 viruses with an E to K mutation at PB2-627 may have increased since the Qinghai Lake (China) outbreak in free-living birds. Virtually all viruses isolated from this outbreak in free-living birds and poultry including chickens, domestic ducks and geese also had PB2-627K [17]. These H5N1 viruses, characterized by the presence of the clade 2.2 HA gene [18], spread to Southern Asia, Africa and Europe and almost invariably contain PB2-627K [19].

In contrast to the body of studies in mammalian species there are relatively few studies comparing the pathogenesis of H5N1 AIV in ducks and chickens or assessing the contribution of specific amino acids to the pathogenicity in avian species. Infection of ducks with different H5N1 isolates showed differences in virus replication and pathogenicity [19]–[23]. Differences in pathogenicity in chickens have been linked to NP, PB1 and PB2 genes [24]–[25] and within the NP gene a change from A to K at position 184 increased virus titers and reduced the mean time to death [26]. Hulse-Post *et al.* [27] examined highly virulent large plaque and nonpathogenic small plaque isolates purified from A/Vietnam/1203/2004 H5N1 (VN/1203) for sequence differences. In ducks, the pathogenicity was associated with differences in PB1 and PA.

One of the major differences between chickens and ducks infected with HP H5N1 viruses is the difference in total mortality and the mean time to death [21], [28]–[30]. Recently, Barber *et al.* [31] suggested that the absence of RIG-I in chickens makes them more susceptible to HP H5N1 infection and may explain the greater pathogenicity seen in chickens compared to ducks which express RIG-I. H5N1 replication in endothelial cells of infected chickens has been associated with high mortality and shortened mean time to death as compared to ducks [25], [29]. Furthermore, we have previously observed that inflammatory cytokine mRNAs are increased significantly within 24 hours post infection (pi) [32]. We speculated that a rapid overwhelming inflammatory response may play a role in the pathogenesis in chickens. Sterz and Weiss [33]–[34] had previously shown that AIV replicates in chicken thrombocytes *in vivo* and *in vitro*. Chicken and duck thrombocytes are highly phagocytic [34]–[37], express low levels of CD154 [38] and after *in vitro* stimulation with LPS can rapidly increase IL-1β, IL-6 and IL-12 mRNA expression [39]–40. Furthermore, chicken thrombocytes release high levels of functional IL-6 following LPS stimulation (Schat, unpublished data).

In this study, we used reverse genetics to generate HP VN/1203 viruses that contain either PB2-627K or PB2-627E. We also produced isogenic low pathogenic (LP) VN/1203 viruses that lacked the hemagglutinin (HA) cleavage site for HP. To our knowledge studies comparing the pathogenicity of such viruses have not been reported in chickens or ducks. Here we report that the switch from K to E at PB2-627 of HP VN/1203 does not influence the pathogenesis in ducks and chickens. However, the PB2-627E mutation in LP VN/1203 increased virulence relative to PB2-627K. Furthermore, we present evidence to support our hypothesis that thrombocytes may play a major role in the pathogenesis of HP H5N1 in chickens, but not in ducks.

## Results

### Virus shedding and transmission

To determine if the change of PB2-627 from E to K in VN/1203 influences horizontal transmission and/or pathogenicity, we infected chickens and ducks with HP VN/1203/K and HP VN/1203/E viruses. On the following day, naïve birds were placed in cohabitation with the inoculated animals. Infection was measured by survival to 14 days pi, isolation of virus from swabs and tissues, and seroconversion.

Chickens infected with HP VN/1203/K or HP VN/1203/E viruses died within 18–24 hours pi. There were no significant differences in replication between the two viruses. Virus was isolated from all organs and oral and cloacal swabs at the time of clinical disease or death and most of the swabs collected at 24 hours pi were positive (Table 1). Virus was not isolated from chickens that subsequently survived until 14 days pi nor were they seropositive, indicating they did not receive an infectious dose. Contact transmission from infected chickens was not established at all for HP VN/1203/E and at a low level with HP VN/1203/K with only 3/14 birds positive (Table 1). All inoculated and contact-challenged ducks became infected as determined by disease or seroconversion in survivors.

**Table 1:** Experimental infection of chickens by inoculation or contact challenge with HP VN/1203/K and HP VN/1203/E.

Virus	Virus	No	Virus isolation: Positive/Total diseased birds^B^								No of
	Challenge^A^	Diseased/Total	24 hrs Post		At Termination						Survivors at 14
			Challenge								Days pi^C^
			O	C	O	C	S	B	L	H	
VN/1203/K	Inoculated	6/7	4/6	3/6	6/6	6/6	6/6	6/6	6/6	6/6	1
	Contact	3/14	3/3	2/3	3/3	1/2^D^	3/3	NT	NT	NT	11
VN/1203/E	Inoculated	5/6	3/5	4/5	5/5	5/5	5/5	4/4^D^	5/5	5/5	1
	Contact	0/14	NT	NT	NT	NT	0/14	NT	NT	NT	14

A Birds were inoculated with 10^5.2^ EID_50_ virus and uninfected birds were placed in contact 24 hrs later.

B O = oral swab, C = cloacal swab, S = spleen, B = brain, L = lung, H = heart. Samples were obtained from dead birds or infected birds euthanized when diseased.

C All survivors at 14 days pi were negative for virus isolation from spleen and for HI antibodies.

D Sample missing from one bird.

To determine the virulence for ducks and the extent of horizontal spread to contact ducks and chickens, ducks were infected with 10^5.8^ and 10^6.0^ EID_50_ of HP VN/1203/E and HP VN/1203/K, respectively. Inoculation with either virus resulted in similar rates of clinical disease in ducks and in chickens, resulting in euthanasia or death in some ducks and all chickens. Virus was readily isolated from oral swabs from all inoculated ducks (Table 2) and all surviving ducks were seropositive. Both viruses were transmitted to 100% of contact ducks. Ducks infected with HP VN/1203/E transmitted the infection to contact ducks more efficiently than HP VN/1203/K infected animals, as evidenced by virus isolation from oral swabs collected after 5 days in cohabitation (p<0.05, two-tailed Fisher's exact test) (Table 2). The mortality rates and all other data were not significantly different between the two contact-infected duck groups. All contact infected chickens died or were euthanized between 5 and 7 days post contact with both viruses.

**Table 2:** Infection of ducks and chickens by inoculation or contact with HP VN/1203/K (K) and HP VN/1203/E (E).

Challenge				Virus isolation from oral swabs: No Pos/Total						Disease^B^			Mean
Virus	EID_50_	Species	No	at Day PI^A^						Mortality	MTD	No with	HI titer
				2	3	4	5^C^	6	7		(days)	Lesions/Survivors	
K	10^6.0^	Duck	12	5/12	12/12	8/12	2/12^a^	2/12	1/11	3/12	7.0	8/9	89
	Contact	Duck	10	…^D^	…	2/10	2/10^a^	0/8	0/8	3/10	6.3	…	110
	Contact	Chicken	5	…	…	…	…	…	…	5/5	6.8	NA^D^	NA
E	10^5.8^	Duck	11	2/11	11/11	10/11	1/11^a^	0/11	0/9	3/11	6.3	6/8	124
	Contact	Duck	9	…	…	4/9	8/9^b^	1/8	0/8	5/9	7.0	…	96
	Contact	Chicken	5	…	…	…	…	…	…	5/5	7.0	NA	NA
None	NA	Duck	10	…	…	…	…	…	…	0/10	NA	0/10	<4

A Virus isolation from oral swabs in Vero cells. The days pi for the contact-infected birds reflect the days post placement.

B Diseased birds were euthanized when clinical signs developed and are included in mortality and mean time to death (MTD). MTD for contact-infected ducks is calculated from the time of placement. The total lesions include histopathology at termination.

C Virus isolations at 5 days pi were significantly different between the contact-infected groups as indicated by different minor case superscript letters (two-tailed Fisher's exact test).

D Not tested (…) or not applicable (NA).

### Clinical disease and pathology in chickens

**Figure 1:**
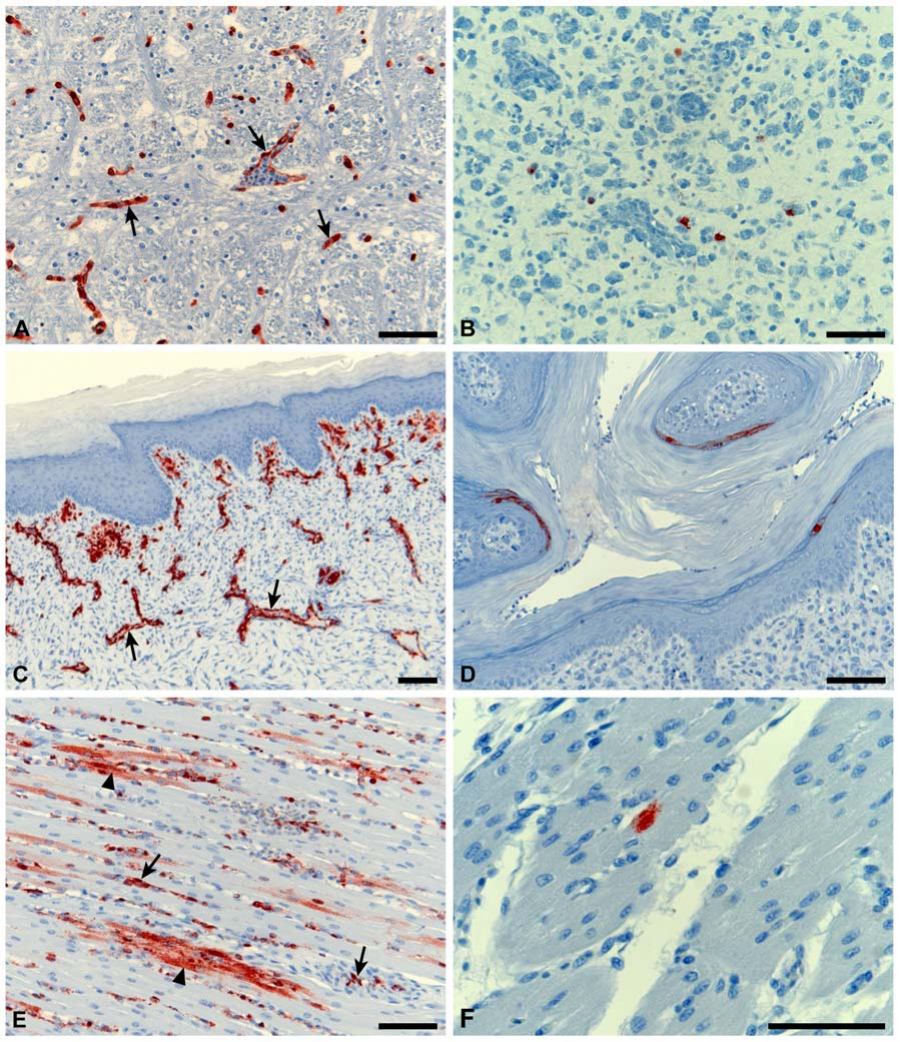
Virus antigen expression in tissues of infected chickens. Chickens were infected with HP VN/1203/E (A, C, E) and LP VN/1203/E (B, D, F). Tissues were taken at 18–36 hours pi from HP infected birds and at 5–13 days pi from LP infected birds. Sections were prepared from brain (A, B), skin from the comb (B, C) and heart (E, F) and stained for the presence of viral antigen. A. Brain, showing high levels of antigen in capillaries (arrows) and in occasional neurons. B. Brain, 13 days pi, showing antigen in neurons within a focus of glial proliferation. C. Skin of comb, showing antigen in capillaries (arrows) and surrounding connective tissue within the dermis. D. Skin of comb, 13 days pi, showing viral antigen in the stratum granulosum. E. Heart, showing antigen predominantly in capillaries (arrows) and in the myocardium (indicated by arrow heads). F. Heart, 5 days pi, showing viral antigen in a single myocardial fiber. All scale bars are 100 µm.

The clinical disease and pathology at the time of death were essentially similar in chickens infected with the HP VN/1203/E and VN/1203/K viruses. Establishment of infection caused mortality or severe depression between 21 and 46 hours pi. The morbidity periods were very short, with some chickens requiring euthanasia within 3 hours of the initial manifestation of clinical signs. There was an absence of gross pathology. Microscopically, there were small foci of necrosis, most noticeably in the red pulp of the spleen, but also in the lung interstitium and lamina propria of the intestine. Tissues of infected birds were assessed for viral antigen by immunohistochemistry at time of euthanasia. High levels of antigen expression was detected mainly in the endothelium (Figure 1A, C, E), indicating that viral replication occurred in all vascularised tissues. Antigen was particularly dense in rich vascular networks, such as in the spleen, lung, glomerular tufts and lamina propria of intestinal villi. Dense antigen was also present in inflammatory foci in the submucosa of various tissues, such as intestine, respiratory airways, liver and kidney. The parenchyma of organs contained low to moderate levels of antigen, and this was most frequent in myocardium (Figure 1E), but also in small foci in the brain, lung, liver, pancreas and kidney tubules.

### Clinical disease and pathology in ducks

There were also no noticeable differences in the clinical disease and pathology between ducks infected with the HP VN/1203/E and VN/1203/K viruses. Gross lesions in ducks were often absent or subtle and included mild pale streaking on the heart, pale or red spots on the pancreas and mildly increased fluid in the body cavities. Microscopic lesions included acute, diffuse, mild to severe cardiomyopathy, diffuse mild to moderate non-suppurative encephalitis and acute focal necrosis of the pancreas. Influenza virus antigen was detected in the myocardium, in foci of neurons in the brain, in skeletal and smooth muscle, necrotic foci in the pancreas (Table 3) and in air sac epithelium. Antigen was rare in lung interstitium and kidney tubules. The pathology observed in contact-infected ducks was similar to that in inoculated ducks.

**Table 3:** Presence of viral antigen in ducks infected with HP VN/1203/K and HP VN/1203/E virus.

Virus	Route of	No of	Days	No. Antigen Positive (Ave Score of Positive Tissues)^C^				
	Infection	Ducks^A^	pi^B^	Spleen	Lung	Brain	Heart	Pancreas
VN/1203/K	Inoculated	3*	7	0	0	1 (2)	1 (2)	0
	Contact	3*	6.3	3 (1.7)	1 (1)	3 (2.7)	3 (2)	2 (2)
VN/1203/E	Inoculated	3*	6.8	0	0	3 (2)	3 (1.7)	2 (1.3)
	Contact	5*	6.3	0	1 (1)	3 (1.7)	3 (2)	1 (2)
VN/1203/K	Inoculated	6	3	0	2 (1)	3 (1.3)	4 (1.3)	0
		6	4	0	2 (1.5)	1 (2)	4 (1.3)	1 (1)
		6	6	0	0	1 (1)	2 (1.5)	0
VN/1203/E	Inoculated	6	3	0	1 (1)	3 (1.7)	6 (1.7)	1
		6	4	0	0	5 (1.4)	6 (1.8)	2^D^(1)
		6	6	0	0	1 (1)	4 (1.7)	0

A The ducks with an asterix are obtained from the experiment described in Table 2. The ducks without an asterix are from the pathogenesis experiment.

B The days pi reflect the mean time to death for the ducks with asterix or the time of euthanasia.

C Number of samples positive for virus antigen as measured by immunohistochemistry. Scoring system: 1 = Antigen sparse, 2 = Antigen common, 3 = Antigen abundant.

D Only 5 samples available.

### Absence of genetic changes following infection and transmission

To determine whether the PB2-627 amino acid had changed during the course of infection *in vivo* we sequenced the corresponding region of viruses recovered from two diseased (for HP VN/1203/E and HP VN/1203/K viruses) contact ducks. In both instances the same sequence as the inoculated virus was obtained suggesting that this position was not undergoing rapid host-driven selection *in vivo*.

### Pathogenesis of infection and disease

**Figure 2:**
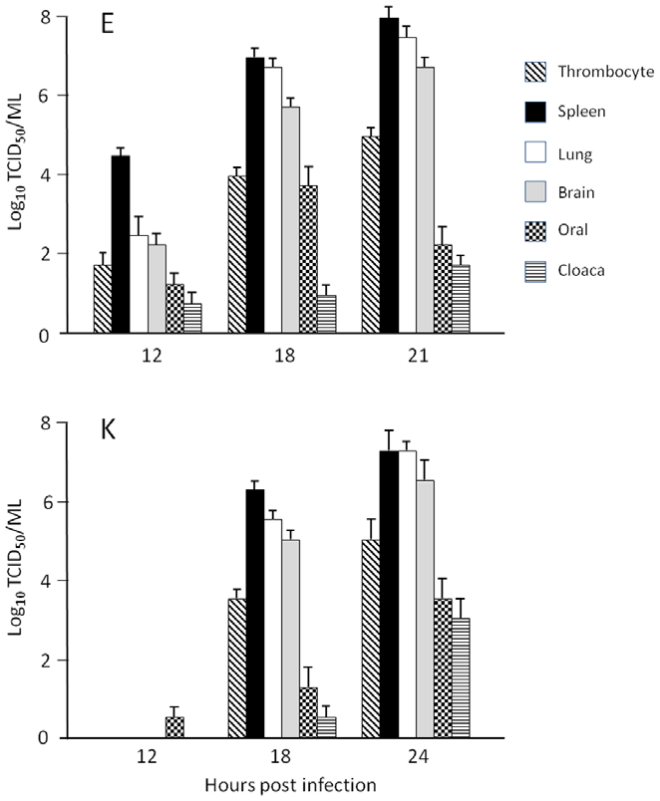
Virus isolation from tissues of infected chickens. Virus titers in thrombocytes, spleen, lung and brain samples, and from oropharyngeal (oral) and cloacal (cloaca) swabs collected from chickens infected with HP VN/1203/E and HP VN/1203/K at 12, 18 and 21 or 24 hours pi. All titrations were performed in Vero cells. Data show mean and SEM of 5–7 birds per time point for each virus.

In order to determine the influence of amino acid substitution at PB2-627 on the pathogenesis of infection, chickens and ducks were inoculated with virus and euthanased at intervals for sample collection. Two experiments were conducted in chickens, both with similar dose and route of inoculation, but sampled at different time points. In the first experiment chickens were inoculated with 10^6.0^ TCID_50_ of either virus. Neither HP VN/1203/E nor HP VN/1203/K were isolated from spleen, lung, and brain tissue samples as well as cloacal and oral swabs collected at 6 and 12 hours pi, however, all specimens collected at 24 hours pi were positive. In the second experiment, chickens infected with 10^6.0^TCID_50_ of HP VN/1203/E had virus in different organs at 12 hours pi in contrast with birds infected with the same dose of HP VN/1203/K (Figure 2). At 18 hrs pi, titers in the different organs and swabs were not significantly different between the two viruses. All tissue samples and swabs contained infectious virus by the time clinical disease developed (21–24 hours pi), with titers between 10^2^ to 10^8^ TCID_50_/mL (Figure 2).

Antigen was not detected in any of the chickens at 6 and 12 hours pi, but was present in all birds sampled at or after 18 hours pi (data not shown). There was no difference in antigen levels between the two viruses at these time points and at time of clinical disease.

With both HP VN/1203/E and HP VN/1203/K virus antigen was detected in duck tissues with peak levels found between 3 and 4 days pi, with a decline in antigen levels by day 6 (Table 3). There was no apparent difference in the tissue tropism between the two viruses with heart and brain most frequently positive. There appeared to be a higher overall prevalence of heart and brain infection in ducks infected with HP VN/1203/E, but the overall differences with HP VN/1203/K were not significant.

**Figure 3:**
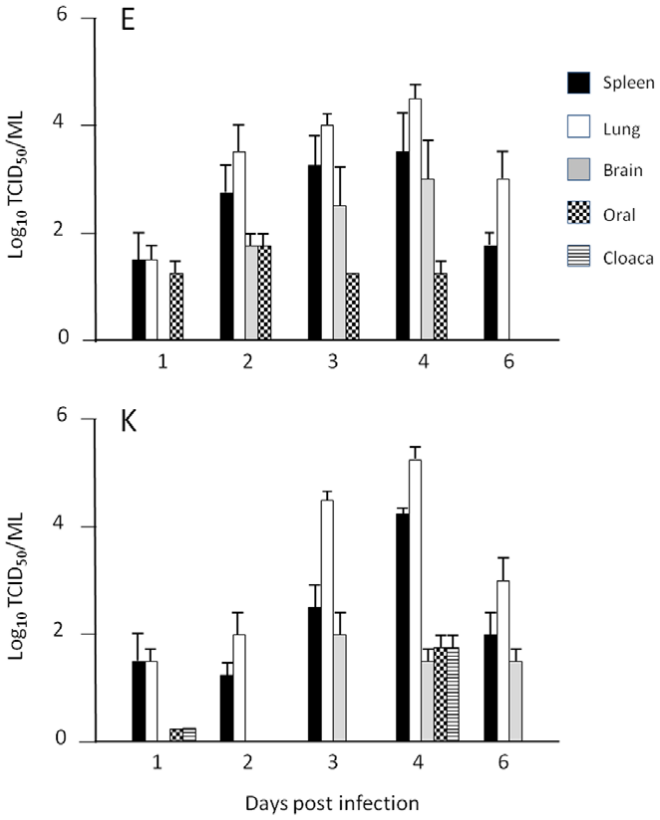
Virus isolation from tissues of infected ducks. Virus titers in spleen, lung and brain samples and from oropharyngeal (oral) and cloacal (cloaca) swabs collected from ducks infected with HP VN/1203/E and HP VN/1203/K at 1, 2, 3, 4, and 6 days pi. All titrations were performed in Vero cells. Data show mean and SEM of 6 birds per time point for each virus.

Virus was isolated from all ducks in both virus groups at 3 and 4 days pi, but not all organs were positive. Titers in the spleen, lung, brain and cloacal and oral swabs at the different time points are presented in Figure 3. Although there were minor differences between the two virus groups all time points, the differences were not statistically significant. Taken together, these findings suggested that in this model there are no significant differences in the kinetics of virus infection and virus distribution in the major organ systems of chickens and ducks as a result of the change of amino acid at position 627 of PB2.

### Infection of chickens with low pathogenic viruses lacking polybasic cleavage site in the HA

The virulence of wild-type HP VN/1203 virus in chickens was overwhelming and precluded detection of moderate differences between PB2-627K and PB2-627E infections. Therefore, we developed isogenic viruses with a deletion of the multibasic cleavage site of the HA; i.e. LP VN/1203/E and LP VN/1203/K. To confirm that the

 LP VN/1203/K and LP VN/1203/E viruses were indeed of low pathogenicity, we inoculated chickens (n = 6) with 10^6.1^ EID_50_ of either virus and evaluated the response for 14 days. Three of the 6 birds infected with LP VN/1203/E developed ataxia and torticollis and were euthanased, one 8 days pi and the other two 13 days pi. These 3 birds had non-suppurative encephalitis and dermatitis, as described below, and virus antigen associated with lesions. While 5/6 chickens seroconverted after infection with LP VN/1203/K, none showed clinical signs (Table 4). The remaining birds of both groups were euthanased and sampled at 14 days pi; although they survived apparently healthy, many had microscopic lesions in brain and skin. Characteristic lesions that were associated with antigen included focal glial nodules with the presence of mononuclear cell perivascular cuffing in the brain and focal dermal and subdermal necrosis, particularly in the comb, and folliculitis. In the absence of viral antigen in such lesions were considered highly likely as indicative of prior influenza virus replication. Such lesions were present in the remaining three chickens that were euthanased at 14 days pi for LP VN/1203/E and in 1 of 5 chickens surviving to 14 days pi for LP VN/1203/K (Table 4). Viral antigen was present in single neurons in glial nodules in the brain (Figure 1B), and at the stratum granulosum of epidermis of skin (Figure 1D) and feather follicles. The incidence of histopathologic lesions in the brain and skin were significantly higher in birds infected with LP VN/1203/E than with LP VN/1203/K (p<0.05, two-tailed Fisher's exact test, Table 4). All birds that survived until termination developed HI antibodies except one bird infected with LP/VN1203/K. However, the antibodies failed to completely clear the virus based on immunohistochemistry and did not prevent the development of clinical disease in the case of the two birds with nervous symptoms at 13 days pi. To confirm that both LP VN/1203/E and LP VN/1203/K viruses replicated *in vivo*, oral secretions collected at 6 days pi were analyzed by RT-PCR; viral RNA was detected in both groups of chickens.

**Table 4:** Clinical, pathological and virological responses in chickens inoculated with LP VN/1203/K or LP VN/1203/E and monitored for 14 days pi^A^.

Virus	Clinical	IHC: No Pos/Total^B^^,^ C		Histopathology: No with Lesions/Total^C^				HI
	Disease	Skin	Brain	Skin	Brain	Pancreas	Total	Antibodies
K	0/6^a^	0/6^a^	0/6^a^	1/6^a^	0/6^a^	0/6^a^	1/6^a^	5/6
E	3/6^a,^ D	4/6^a^	3/6^a^	6/6^b^	5/6^b^	4/6^a^	6/6^b^	6/6

A Birds were euthanized at 14 days pi unless otherwise noted.

B IHC = Immunohistochemistry. Positive samples had sparse antigen in the different organs ( = score of 1).

C Values within a column with a different superscript minor case letter are significantly different at p<0.05 (two-tailed Fisher's exact test).

D Three of the six birds infected with LP VN/1203/E developed ataxia and torticollis and were euthanased, one at 8 days pi and two at 13 days pi.

Chickens infected with 10^6.1^ EID_50_ of LP VN/1203/E and LP VN1203/K were sacrificed at 1, 2, 3, 4, and 5 days pi and organs were examined for the presence of viral antigen by immunohistochemistry and virus isolation by inoculation of 9- to 10-day old embryonated chicken eggs. None of the chickens showed clinical signs, although some sampled at 4 and 5 days pi had lesions, including glial nodules in the brain and mild, focal necrosis in the dermis. Overall, virus isolation rates were higher for LP VN/1203/E than for LP VN/1203/K. The difference was significant at 3 days pi (p<0.05, two-tailed Fisher's exact test, Table 5).

**Table 5:** Pathogenesis of infection with LP VN/1203/K and LP VN/1203/E in chickens.

Virus	Day pi	No. Ag+ (Ave Score of Positive Tissues)^A^				Virus Isolation: No Pos/Total^B^				
		Heart	Brain	Skin^C^	Other^D^	Spleen	Lung	Brain	Throm^E^	Total^F^
K	1	0/6	0/6	0/6	0/6	1/6	0/6	0/6	0/6	1/6
	2	0/6	0/6	0/6	0/6	0/6	1/6	0/6	0/6	1/6
	3	0/6	0/6	0/6	0/6	0/6	0/6	0/6	0/6	0/6*
	4	0/6	1/6 (1)	1/6 (1)	1/6 (1)	2/6	1/6	2/6	0/6	2/6
	5	0/6	0/6	0/6	0/6	0/6	0/6	2/6	0/6	2/6
E	1	0/6	0/6	0/6	0/6	1/6	1/6	0/6	0/6	2/6
	2	0/6	0/6	0/6	0/6	0/6	1/6	0/6	0/6	1/6
	3	1/6 (1)	0/6	1/6 (1)	1/6 (1)	5/6	4/6	5/6	0/6	6/6*
	4	1/6 (1)	1/6 (1)	1/6 (1)	1/6 (1)	1/6	1/6	1/6	1/6	3/6
	5	2/6 (1)	1/6 (1)	2/6 (1)	2/6 (1)	1/6	3/6	1/6	0/6	3/6
UC	1+5	0/6	0/6	0/6	0/6	0/12	0/12	0/12	0/12	0/12

A Number of samples positive for virus antigen as measured by immunohistochemistry. Scoring system: 1 = Antigen sparse, 2 = Antigen common, 3 = Antigen abundant.

B Virus isolation was performed by inoculation of 3 embryonated chicken eggs per tissue sample.

C Includes dermis of feathered skin, feather pulp and comb; for other tissues see the result section.

D Other tissues are liver, kidney tubules, spleen, lung interstitium, connective tissues, lymphoid follicles, and salivary glands.

E Thrombocytes.

F Number of birds with one or more positive tissues.

*Significantly different from each other (P<0.05, two-tailed Fisher's exact test).

Viral antigen was detected in neurons and glial cells (with or without associated lesions), in single myocardial fibers (Figure 1F), kidney tubular epithelium, single cells in dermis, connective tissues (follicle sheath, periosteum), nasal gland and feather pulp. Definitive antigen in endothelium, as seen with the HP AIV infections, was not found. Interestingly, LP VN/1203/E caused a higher incidence of virus-antigen positive tissues than LP VN/1203/K starting at 3 days pi. The difference between the total number of antigen positive tissues of all birds combined for 3–5 days pi is significantly higher for LP VN/1203/E (14/18) than LP VN/1203/K (3/18) (p<0.05, two-tailed Fisher's exact test).

Sequence analysis confirmed that the virus obtained from the bird with neurological signs at 8 days pi and from the four birds that we viral-antigen positive still retained the HA cleavage site associated with the LP form as well as the original E at PB2-627.

In conclusion, these studies indicated that there are differences associated with infection in chickens between viruses with E *versus* K at PB2-627, but that these changes can only be demonstrated when birds were infected with the LP viruses.

### Thrombocyte infection with HP and LP viruses

Previous studies by Sterz and Weiss [33]–[34] suggested that AIV can replicate in thrombocytes, we therefore examined whether thrombocytes obtained from infected chickens and ducks contained infectious virus. Thrombocytes from all chickens contained infectious virus at 18 and 21–24 hours pi with either HP VN/1203/E or HP VN/1203/K, and at 12 hours pi for 5/6 chickens infected with HP VN/1203/E (Figure 2). In ducks, thrombocyte preparations from only some of the animals contained infectious virus but in contrast with the findings in chickens the titers were extremely low (≤30 TCID_50_, data not shown). In contrast, thrombocytes from chickens infected with LP VN/1203/E and LP VN/1203/K were negative for virus isolation except for one sample from the LP VN/1203/E group taken at 4 days pi.

Both HP VN/1203/E and VN/1203/K viruses could be isolated at high levels from chicken thrombocytes suggesting that AIV might replicate in these cells or serve as a vehicle for the spread virus in the vasculature. To quantify the viral RNA in thrombocytes, we analyzed purified cells by real-time RT-PCR using primers specific for viral genomic RNA. Chicken thrombocytes collected at 18 hours pi with HP VN/1203/K or HP VN/1203/E had Ct values of 18.6 and 17.0 for genomic RNA and 17.8 and 16.2 for cRNA specific cDNA reactions, respectively. In contrast thrombocytes from uninfected birds had Ct values of >40 for both primers.

The profound transcriptome changes observed in thrombocytes collected at 18 hours pi suggested that HP VN/1203/K and HP VN/1203/E may have initiated replication in chicken thrombocytes or progenitor megakaryocytes. Table 6 shows the complete list of significant changes, defined as ≥2-fold increase in transcription relative to uninfected controls. Analysis of the TLR receptor signaling pathway indicated significant upregulation of TLR3, which can be triggered by dsRNA, and involve STAT1, IRF7, and IFNAR2. In addition several cell adhesion molecules (SELE, VCAM1, CD274), SOCS 1 and MX were also highly upregulated in thrombocytes.

**Table 6:** Two-fold or more upregulated selected genes in thrombocytes 18 hours pi of chickens with HP VN/1203/K and HP VN/1203/E relative to uninfected controls.

Pathway name	Gene symbol	P-value	Fold change	Gene Description
Adipocytokine signaling pathway	SOCS3	0.0491	2.085	Suppressor of cytokine signaling 3
Insulin signaling pathway	SOCS3	0.0491	2.085	Suppressor of cytokine signaling 3
	SOCS1	0.0021	6.154	Suppressor of cytokine signaling 1
Toll-like receptor signaling pathway	STAT1	0.0006	3.637	Signal transducer and activator of transcription 1, 91 kDa
	TLR3	0.0309	2.256	Toll-like receptor 3
RIG-I-like receptor signaling pathway	TRIM25	0.0329	2.037	Tripartite motif-containing 25
	TMEM173	0.0042	2.219	Transmembrane protein 173
	IFIH1	0.0013	5.595	Interferon-induced with Helicase C domain 1
Apoptosis	CASP7	0.0089	2.205	Caspase 7, apoptosis-related cysteine peptidase
Cell adhesion molecules (CAMs)	SELE	0.0209	2.311	Selectin E (endothelial adhesion molecule 1)
	VCAM1	0.0011	8.181	Vascular cell adhesion molecule 1
	CD274	0.0081	4.487	CD274 molecule
Interferon stimulated genes	MX	0.0168	5.175	Chicken MX gene

These results showed a major difference in the response of thrombocytes between chickens and ducks inoculated with HP AIV and between chickens inoculated with HP *versus* LP viruses. The microarray results further support the postulated importance of thrombocytes for the pathogenesis and pathology of HP virus in chickens.

## Discussion

Several previous studies have compared the pathogenicity of different HP H5N1 isolates in chickens and ducks. Most of these isolates caused peracute mortality in chickens often without clear pathology [30], [41]–[42]. The pathogenicity in domestic ducks varied from 0 to 100% depending on the isolate [19], [22]–[23], [43]–[44], age at infection [45]–[46], and virus dose [47]–[48]. The pathogenicity determinants for these viruses have only been partially elucidated. In addition to the HA cleavage site, the PA, PB1, PB2 and NP genes contribute to differences in the pathogenicity of HP H5N1 viruses in chickens and ducks [24]–[27], [49]–[50]. In contrast to the many studies showing the importance of the K versus E at PB2-627 in mammalian species, there is a lack of information on this change for the pathogenesis in chickens and ducks. Our studies show that infection with HP VN/1203/K and HP VN/1203/E are both lethal in chickens, causing rapid death. The absence of antibodies in chickens that survived inoculation with 10^4.5^ to 10^5.2^ EID_50_ confirmed a previous report indicating that the minimum infectious dose of wild type Asian lineage H5N1 viruses for chickens is relatively high, 10^3.4^ EID_50_ for ID_50_, and almost identical to the lethal dose [47]. HP VN/1203/K and HP VN/1203/E were also equally pathogenic for white Pekin ducks with 25–55% disease in inoculated and contact-infected ducks, respectively, using a moderate dose of 100 EID_50_ and 5-week-old ducks. Virus isolation from the oral swabs obtained from the contact-infected ducks suggested subtle differences in replication between the two viruses, but the subsequent time course experiment to evaluate the pathogenesis in ducks failed to reveal significant differences between the two viruses. Clearly, the clade 2.2 viruses of the Qinghai Lake outbreak with PB2-627K can be as pathogenic as viruses with PB2-627E and spread horizontally just as readily as other H5N1 viruses among birds [17], [19], [51]–[62].

Infection of chickens with 10^5.9^ EID_50_ of HP VN/1203/K and HP VN/1203/E showed that virus was being shed at low levels as early as 12 hours pi and HP VN1203/E but not HP VN1203/K was isolated from respiratory and systemic site samples. Antigen from both viruses was abundant in these organs as early as 18 hours pi reflecting rapid replication leading to fatal outcomes between 21 and 24 hours pi. The rapid systemic dissemination of virus in chickens is consistent with earlier reports [25], [42] and contrasts with the slower dissemination of the HP VN/1203/E and HP VN/1203/K viruses in ducks.

In addition to the difference between chickens and ducks in extrapulmonary virus spread and replication kinetics, two other major differences were observed. First, the distribution of viral antigen in tissues was different between the two species. Vascular endothelial cells were positive for viral antigen at 18 hours pi with HP VN/1203/E and HP VN/1203/K in the tissues examined from infected chickens as has been reported by others [25], [29]–[34], [42], [63]. In contrast endothelial expression of viral antigen in ducks was rare [29]. The second major contrast between chickens and ducks was the striking difference in the virus titers in thrombocytes. The high virus titers associated with the chicken thrombocytes as well as the detection of viral RNA clearly indicated the presence of virus in the purified cell preparations. Minor contamination with other monocytes and heterophils cannot be excluded but seems unlikely. We cultured thrombocytes for 24 hours and found that the cultures consisted of >95% thrombocytes (Schat and Guo, unpublished data). It is currently unknown whether the HP AIV was phagocytosed by the thrombocytes or that the virus entrance was receptor-mediated. The finding that duck thrombocytes were basically negative for virus between 1 and 6 days pi is of interest in view of the differences in pathology and viral dissemination.

The question of whether the pathogenicity in chickens is influenced by the change of K to E at PB2-627 could not be resolved in studies using the wild-type HP VN/1203 virus pair due to the overwhelming pathology in this species. However, LP virus counterparts with a deletion of the HA multibasic cleavage site clearly showed that the presence of E at PB2-627 had a significant impact on the pathology. LP VN/1203/E did spread systemically, but at low levels and without causing high lethality, as reported previously [64], and in contrast to its HP counterpart, was not detected in thrombocytes. Absence of virus in thrombocytes may allow for a more effective host antiviral response. The mechanisms mediating increased pathogenicity in LP VN/1203/E as compared to LP VN/1203/K are not clear. Sequence analysis of virus re-isolated from inoculated and contact-infected animals confirmed that the HA and PB2 gene had not changed and were identical to the inoculated viruses. Pillai *et al.* [65] showed that infection of chickens with a diverse collection of LP AIV isolates caused minimal lesions. However, Mundt *et al.* [66] found some mild lesions in some organs including the cerebrum after infection with North-American lineage viruses such as A/chicken/Pennsylvania/13609/93 (H5N2) and A/chicken/Texas/167280-4/02, suggesting that systemic infection can occur with LP AIV. The use of HP AIV isolates in which the HA cleavage site has been mutated to the LP cleavage site may be advantageous to further identify virulence factors for the Asian H5N1 strains.

Involvement of thrombocytes in the infection is also suggested by the microarray data. TLR3 and several of the genes involved in the TLR signaling pathway were significantly upregulated during infection. Moreover, SOCS1, which can regulate TLR-mediated signal transduction [67] was also strongly upregulated. Le Goffic *et al.* [68] showed that influenza A viruses activate TLR3-dependent proinflammatory responses in epithelial cells, leading to increased levels of IFN-β production [69]. We have recently reported that HP VN/1203/K, but not LP VN/1203/K, caused a strong upregulation of IL-6, IL-12, IFNα, IFNβ, IFNγ and IFNλ mRNA at 24 hours pi [32]. It is certainly feasible that infection of thrombocytes plays a major role in the rapid upregulation of the proinflammatory cytokines. It is also of interest to note that several cell adhesion molecules were upregulated. Some of these molecules such as VCAM1 and selectin E may bind to neutrophils leading to the formation of micro-thrombi which are often associated with HPAIV infection [41]. Clearly, additional studies are needed to further elucidate the role of thrombocytes in the pathogenesis of HP AIV in chickens. Moreover, the correlation of viral load differences in chicken and duck thrombocytes with differential species-specific susceptibility between these animals suggests a possible role in pathogenesis.

In conclusion, our studies indicated that HP viruses with PB2-627E may be marginally more pathogenic in ducks than viruses with PB2-627K, but such differences could not be demonstrated in chickens due to the rapid onset of mortality. However, the LP VN/1203/E was certainly more pathogenic in chickens than its PB2-627K counterpart. Finally, our studies indicate that thrombocytes may play a role in the pathogenesis of HP Asian H5N1 AIV infections.

## Materials and Methods

### Animal Ethics Statement

This study was carried out in strict accordance with the recommendations in the National Health and Medical Research Council's Australian Code of Practice for the Care and Use of Animals for Scientific Purposes 7^th^ edition. All experimental protocols were approved by the CSIRO Australian Animal Health Laboratory Animal Ethics Committee (Approval numbers AEC 1152, 1174, 1300 and 1301) at the Australian Animal Health Laboratory (Bureau of Animal Welfare Scientific Procedures Premises Licence SPPL 113). All efforts were made to minimize suffering.

### Animals, husbandry and biocontainment

Four-week-old, straight-run broilers were obtained from a commercial producer. Five-week-old White Pekin ducks were obtained from Luv-a-Duck (Victoria, Australia). All birds were identified individually with leg bands and were assigned to the different treatment groups for each experiment using a randomization tool (www.graphpad.com). Birds were housed in Microbiological Containment level 3 Zoonotic rooms with the temperature set at 22°C and the airflow set at approximately 15 air changes/hour [23].

### Virus strains

HP VN/1203/K was derived from the parental wild-type virus by standard reverse genetics methods [70]. Mutations at positions 627 of the PB2 were generated by standard oligonucleotide mutagenesis methods [71] to generate HP VN/1203/E. An additional mutation was generated in both VN/1203/K and VN/1203/E by deleting 4 basic amino acids in the HA cleavage site. These LP viruses are referred to as LP VN/1203/K and LP VN/1203/E, respectively. All viruses were propagated by two passages in 10-day-old embryonated specific-pathogen-free chicken eggs and stored at −80°C until use. Virus stocks were titrated by endpoint dilution to determine the EID_50_. Following propagation, the full genomes of rescued viruses were sequenced to confirm the presence of the appropriate wild type or mutant sequence.

### Virus challenge

Birds were inoculated by placing one or two drops of virus in each eye and nostril, and instilling the remainder into the oral cavity. Virus titers were confirmed by back titration in 9- to 10-day-old embryonated chicken eggs.

### Sample collection for virus isolation

Pharyngeal and cloacal swabs were collected into 2 mL of phosphate-buffered saline containing penicillin, streptomycin and gentamycin. After euthanasia, fragments of spleen, brain, lung and heart tissues were frozen at −80°C until processing for virus isolation. For some experiments, small amounts of 1.0 mm silicon carbide beads (Daintree Scientific, St Helens, Tasmania, Australia) were added to the collection tubes with phosphate-buffered saline.

Thrombocytes were prepared as described by Scott and Owens [39]. Cells were resuspended in 0.2 ml of HBSS and stored at −80°C until use. The dilution factor of plasma in the final cell preparation was estimated to be between 5 and 6 log_10_.

### Virus isolation

Tissue samples were ground by mortar and pestle using sterile sand or pulverized using a MP Fast-Prep24 instrument (MP Biomedicals, Inc, Solon, OH) when collected with silicon carbide beads and diluted in PBS to 10% w/v homogenates. Virus isolations for the transmission experiment in chickens were performed by inoculating 10-day-old embryonated chicken eggs. Allantoic fluids from all eggs were examined for the presence of HA using chicken red blood cells [72]. Tissue extracts and fluids from swabs obtained from birds inoculated with HP AIV virus were titrated directly in Vero cells as described [23] except that low dilutions of samples causing non-specific cytopathic effects (CPE) in cell culture were tested for HA if higher dilutions did not show specific CPE. The limit of detection of HPAI virus in Vero cells was 1.25log_10_ TCID_50_/mL.

### Serology

Blood samples were obtained from all experimental birds prior to challenge and from survivors at termination. Sera were stored at −20°C until used for the hemagglutination inhibition test [72].

### Histopathology and immunohistochemistry

Tissues were collected and processed for histology and immunohistochemistry as described by Bingham *et al.* [23].

### RNA extraction and RT-PCR

Total RNA was extracted from animal tissues using an RNA isolation kit (RNEasy, Qiagen, Doncaster, Australia) according to the manufacturer's instructions. A viral RNA extraction kit, QIAamp Viral RNA Mini Kit, (Qiagen) was used to extract RNA from oral and cloacal swabs according to the manufacturer's instructions. RT-PCR of the HA cleavage site was conducted using the oligonucleotide primers Fwd 5′-TGTCAAGAAAGGGGACTC-3′ and Rev 5′-CTTTGTCTGCAGCGTACC-3′. RT-PCR of the PB2 gene was conducted using the primers Fwd 5′-CCAAAGATTAAACCCCATGCATCA-3′ and Rev 5′-ACATATGGTCTCGTATTAGTAGAAACAAGGTCGTTT-3′ All samples were subjected to electrophoresis and amplicons were visualised and photographed (Kodak Digital Science, Rochester, NY).

### PCR purification and DNA sequencing

PCR products were purified using a QIAquick® PCR Purification Kit (Qiagen, Germany) according to the manufacturer's instructions. DNA sequencing was performed by the sequencing facility at CSIRO-AAHL using an ABI Prism 377 automated DNA sequencer (Applied Biosystems) and the oligonucleotide primers shown above. Nucleotide sequence data and the translated amino acid sequence were subjected to sequence alignments using clone manager 9 (Version 9.0, Scientific and Educational Software).

### Real-time RT-PCR

Real-time RT-PCR to verify the presence of H5N1 was performed to determine if virus was replicating in the thrombocytes, according to the method of Heine *et al.* [73], using the modifications described below. RNA was isolated from the same samples analyzed in the microarray assay, using the Meridian total RNA isolation kit (Cartagen, Seattle, WA). RNA was converted to cDNA using the Quantitect reverse transcription kit (Qiagen), in two separate reverse transcription reactions with either the forward primer AGCAAAAGCAGGTAGATGTTGAA which anneals to viral HA genomic RNA, or the reverse primer AGTAGAAACAAGGTAGTTTTTTACTCC which anneals to HA cRNA. Following reverse transcription each cDNA sample was subjected to real-time PCR to verify the presence of H5N1 viral RNA and cRNA as described [73]. The thermal cycling parameters consisted of an initial Taq polymerase hot start activation at 95°C for 10 min, followed by 45 cycles of 15 sec at 94°C and 1 min at 60°C. Assays were performed on an AB7900HT real time PCR system (Applied Biosystems) and analyzed using the associated sequence detection software version 2.3 (Applied Biosystems).

### Microarray Assay

Total RNA for all samples was isolated using the Meridian total RNA isolation kit (Cartagen, Seattle, WA). One microgram of total RNA was reverse transcribed into cDNA and indirectly labeled with Cy3 using the NimbleGen One-Color DNA labeling kit (Roche, Basel, Switzerland). Each Cy3 labeled cell culture sample was individually hybridized to the NimbleGen whole genome chicken array (designed in-house) and hybridized according to manufactures instructions. The custom array design has been deposited into ArrayExpress (accession number A-MEXP-2133) and all data is MIAME compliant and has been deposited in ArrayExpress (accession number E-MEXP-3432). Arrays were scanned at 2.0 microns on a NimbleGen MS 200 scanner. Raw data were extracted using NimbleScan software v2.1 (Roche). All arrays were subjected to quality control measures before being included in the analysis. The signal intensities from all arrays were normalized using Robust Multiple-chip Analysis (RMA) [74]. Subsequent statistical tests were carried out using Genespring version 7.2 (Silicon Genetics, Santa Clara, CA) to determine all genes differentially regulated at a false discovery rate (FDR) of p = 0.05. Pathway analysis was conducted using Genowiz Version 4.0.5.3 (Ocimum Biosciences, Gaithersburg, MD).

### Experimental design

To determine pathology, viral shedding and transmission, four-week-old broilers or 5-week-old ducks were infected with HP VN/1203/K or HP VN/1203/E. One day later age-matched uninfected chickens or ducks were added to each infected group. Oral and cloacal swabs were collected daily from all birds. Birds were euthanized prior to the development of severe clinical illness and tissue and blood samples were collected for virus isolation, immunohistochemistry and histology. Sera were collected from all surviving birds at the trial termination, 14 days pi.

To study the early development of infection and disease, groups of chickens and ducks were inoculated with HP VN/1203/K and HP VN/1203/E and individual birds were euthanased and sampled at defined time intervals. Chickens were euthanized at 6, 12, 18 and 24 hours pi. Infected ducks were euthanized at 1, 2, 3, 4 and 6 days pi. At each time point, oral and cloacal swabs, spleen, lung and brain tissues and thrombocytes were collected for virus isolation. In addition, tissues were collected for immunohistochemistry and histopathology. Uninfected control birds from the same batch were euthanized at the start of the experiments (chickens) or towards the end (ducks).

Because both of these HP viruses caused disease and or mortality in chickens within 24 hrs, we used LP VN/1203/K and LP VN/1203/E to determine if the presence of E versus K at PB2-627 influences the pathogenicity of infection in chickens. In a pilot trial, infected chickens were observed for 14 days pi to confirm that these viruses were low pathogenicity. Oral swabs were collected from these chickens at 6 days pi and analyzed by real-time RT-PCR. Surviving birds were bled at 14 days pi for serum collection and euthanized. Samples from different organs were collected for immunohistochemistry and histopathology. To further study the pathogenesis of infection with both low pathogenic viruses, chickens were infected and euthanized at 1, 2, 3, 4 and 5 days pi and samples were collected for virus isolation, immunohistochemistry and histology. Uninfected control birds from the same batch were euthanized simultaneously with infected chickens at 1 and 5 days pi.

### Statistical analysis

Where appropriate, the two-tailed Fisher's exact test was used to determine whether numerical differences between virus isolation rates, pathology and immunohistochemistry are significant. Significant differences for all tests were declared at P<0.05.
